# Advanced Oxidation Processes Coupled to Nanofiltration Membranes with Catalytic Fe^0^ Nanoparticles in Symmetric and Asymmetric Polyelectrolyte Multilayers

**DOI:** 10.3390/membranes13040388

**Published:** 2023-03-28

**Authors:** Tao Wang, Enrique Serra Bachs, Joris de Grooth, Wiebe M. de Vos

**Affiliations:** MESA+ Institute of Nanotechnology, University of Twente, 7500 AE Enschede, The Netherlands

**Keywords:** catalytic membranes, in situ synthesis, asymmetric polyelectrolytes multilayers, advanced oxidation processes

## Abstract

The in situ synthesis of Fe^0^ particles using poly-(acrylic acid) (PAA) is an effective tool for fabricating catalytic membranes relevant to advanced oxidation processes (AOPs). Through their synthesis in polyelectrolyte multilayer-based nanofiltration membranes, it becomes possible to reject and degrade organic micropollutants simultaneously. In this work, we compare two approaches, where Fe^0^ nanoparticles are synthesized in or on symmetric multilayers and asymmetric multilayers. For the membrane with symmetric multilayers (4.0 bilayers of poly (diallyldimethylammonium chloride) (PDADMAC)/PAA), the in situ synthesized Fe^0^ increased its permeability from 1.77 L/m^2^/h/bar to 17.67 L/m^2^/h/bar when three Fe^2+^ binding/reducing cycles were conducted. Likely, the low chemical stability of this polyelectrolyte multilayer allows it to become damaged through the relatively harsh synthesis. However, when the in situ synthesis of Fe^0^ was performed on top of asymmetric multilayers, which consist of 7.0 bilayers of the very chemically stable combination of PDADMAC and poly(styrene sulfonate) (PSS), coated with PDADMAC/PAA multilayers, the negative effect of the Fe^0^ in situ synthesized can be mitigated, and the permeability only increased from 1.96 L/m^2^/h/bar to 2.38 L/m^2^/h/bar with three Fe^2+^ binding/reducing cycles. The obtained membranes with asymmetric polyelectrolyte multilayers exhibited an excellent naproxen treatment efficiency, with over 80% naproxen rejection on the permeate side and 25% naproxen removal on the feed solution side after 1 h. This work demonstrates the potential of especially asymmetric polyelectrolyte multilayers to be effectively combined with AOPs for the treatment of micropollutants (MPs).

## 1. Introduction

As a result of the extensive use of pesticides, personal care products, pharmaceuticals, and steroid hormones, among others, various organic micropollutants (MPs) have now been widely detected in surface waters and ground waters [1,2]. Not only are such MPs associated with clear negative effects, such as acute and chronic toxicity, endocrine disruption, and latency carcinogenicity, but it is also clear that current wastewater treatment plants cannot effectively eliminate MPs. New technologies to treat wastewater, thereby removing or degrading the MPs, are needed [3,4]. Recently, catalytic membranes based on advanced oxidation processes (AOPs) have demonstrated great potential to effectively treat wastewater containing MPs [5,6]. The embedded catalysts within the membrane structure can activate oxidants (e.g., peroxymonosulfate, PMS or peroxydisulfate, PDS) to generate hydroxyl radicals, sulfate radicals, or singlet oxygen, which can degrade MPs to smaller organics and eventually completely to carbon dioxide and water [7,8,9,10]. However, for this approach, ultrafiltration membranes are typically used, with pores that are far too large to reject MPs [11,12]. To further integrate membrane separation with AOPs, a catalytic nanofiltration membrane, which can not only degrade but also reject MPs is highly desired.

In the structural design of an AOPs-based catalytic nanofiltration membrane consisting of a porous support and a dense selective layer, the position to introduce the catalysts is important. It determines the amount of required catalysts, the access of reactants to the catalysts, and the residence time for the reactions, in turn significantly influencing the degradation efficiency of the AOPs-based catalytic membranes [13]. Immobilizing catalysts within the porous support is widely used to build catalytic membranes, which can be easily achieved by blending catalysts within the casting solution [5,14]. The small-sized catalysts are uniformly distributed within the polymer matrix after the phase inversion process. Moreover, as the porous support is much thicker compared to the selective layer, both the MPs and oxidants have a long residence time for the catalytic reactions, which is suitable for the degradation efficiency of MPs [15,16,17,18]. However, the blending method unavoidably leads to the coverage of the polymer on the catalysts embedded within the porous support, lowering the accessibility of MPs and oxidants to catalysts [18]. Moreover, the follow-up coating of a selective layer on top of the catalytic support also hinders the access of MPs and oxidants to catalysts. As a result, the degradation efficiency of MPs would decrease. Embedding catalysts on or in the selective layer is an alternative option where the catalysts can directly contact the oxidants and MPs [5]. In this approach, the highly concentrated retentate is also expected to be partly mitigated since the reactive radicals generated on the surface of the selective layer can degrade the MPs while they are rejected by the retentate. Unfortunately, when the catalysts are simply mixed with the monomers or polyelectrolytes that are used to build a selective layer, the aggregation and the nonuniform distribution of the catalysts in the selective layer result in a significant drop in degradation efficiency. Another potential problem is the shorter residence time due to the much thinner selective layer compared to the support, which may not be sufficient for the activation of oxidants. Therefore, a feasible method is desired, which can achieve not only an even distribution of catalysts but also a sufficient quantity of catalysts on the selective layer of membranes to enable sufficiently quick activation.

In situ synthesizing Fe^0^ particles with acrylic acid (AA) has been widely explored [19,20], and the synthesized Fe^0^ particles are shown to efficiently activate PMS in the AOPs [21,22], which provides the possibility of evenly introducing catalysts in the selective layer containing AA molecules. In a typical in situ synthesis process of Fe^0^ with AA, Fe^2+^ ions are first bound to AA through ion exchange and then reduced by NaBH_4_ to form Fe^0^ nanoparticles. Therefore, to perform this in situ synthesis on membranes, immobilization of AA on the membrane surface is required. In the work of Bhattacharyya et al., AA was polymerized on the surface of PVDF membranes via ethylene glycol at 90 °C [23]. With the same crosslinking agent (ethylene glycol), Larissa et al. applied microwave radiation to polymerize AA on the membrane into poly(acrylic acid) (PAA) instead of thermal polymerization [24]. Different from the in situ polymerization of AA on the membrane structure, PAA was used directly in the work of Shi et al., and 1.5 bilayers of PAA and poly-(diallyl dimethylammonium chloride) (PDADMAC) were coated on the membrane with a precoated layer of polydopamine and polyethylenimine (PEI) [25]. Moreover, the parameters of the synthesis, such as the number of bilayers coated, the pH of the polyelectrolyte solution, and the binding/reducing cycles, can be controlled to manipulate not only the amount but also the size of the synthesized Fe^0^ [20]. However, this in situ synthesis of Fe^0^ risks the rejection ability of membranes, as the small-sized Fe^0^ synthesized within the selective layer could make it less dense or even defective. This is especially the case as PAA is a weak polyelectrolyte, only allowing the formation of polyelectrolyte multilayers (PEMs) that have lower chemical stability. Therefore, it is important to explore the rejection ability of polyelectrolyte multilayers containing PAA after the in situ synthesis of Fe^0^. Currently, the reported in situ syntheses of Fe^0^ with PAA have all been conducted on symmetric polyelectrolyte multilayers [20,25,26], where the entire PEM is made up of a single polyelectrolyte couple. In such a system, it is difficult to implement a successful synthesis of catalyst while retaining its original selectivity to MPs.

To circumvent this problem, the building of asymmetric polyelectrolyte multilayers provides a possibility. In such a multilayer, a thin and dense PEM is typically applied to a more open PEM to create very thin active separation layers. Indeed, it has already been reported that the membranes with asymmetric polyelectrolyte selective layers exhibited excellent MPs rejection [27]. For example, relatively thick but open polyelectrolyte multilayers of PDADMAC/poly(styrene sulfonate) (PSS) were first coated to make a porous support membrane defect-free, and then the thin but dense polyelectrolyte multilayers of PAA/poly(allylamine hydrochloride) (PAH) coated on top of the PDADMAC/PSS multilayers provide sufficiency selectivity. The asymmetric PEM structure thus consists of different polyelectrolyte multilayers possessing different functionalities, providing the opportunity of building catalytic membranes with suitable selectivity. With multilayers of PDADMAC/PSS first coated working as the selective layer and in this work, multilayers of PDADMAC/PAA second coated providing carboxyl groups to fix Fe^2+^ ions, it could finally become possible to build catalytic nanofiltration membranes where a synergy of physical separation and AOPs-based chemical degradation can be accomplished. Especially since both PDADMAC and PSS are strong polyelectrolytes, providing the required chemical stability during synthesis and the AOP process.

In this work, the in situ synthesis of Fe^0^ in symmetric polyelectrolyte multilayers and on asymmetric polyelectrolyte multilayers is compared. To synthesize Fe^0^ catalysts on the selective layer, all the catalytic membranes were coated with the outmost layer of PAA. Naproxen and PMS were chosen as the model micropollutant and oxidant, respectively, to measure the degradation efficacy of the catalytic membranes. The membranes with Fe^0^ synthesized on symmetric polyelectrolyte multilayers were first fabricated. Different Fe^2+^ binding/reducing cycles were performed on the membranes with 4.0 bilayers of PDADMAC and PAA to explore the effects of synthesizing Fe^0^ in/on a selective layer on the membrane performances, including permeability, rejections, and degradation efficacy. After that, asymmetric polyelectrolyte multilayers were fabricated by coating 7.0 bilayers of PDADMAC and PSS before the coating of PDADMAC and PAA layers. The effect of the layer number of PDADMAC and PAA on the naproxen treatment efficacy was further explored. This work compares in situ synthesizing Fe^0^ particles on top of a symmetric multilayer and an asymmetric multilayer, demonstrating the asymmetric PEMs as a very relevant platform for high-performance catalytic membranes.

## 2. Materials and Methods

### 2.1. Chemicals

Poly (diallyldimethylammonium chloride) (PDADMAC, 200–350 kDa, 20 wt% in water), poly (acrylic acid) (PAA), poly(styrene sulfonate) (PSS, 200 kDa, 30 wt% in water), Oxone (KHSO_5_ · 0.5KHSO_4_ · 0.5K_2_SO_4_, mono persulfate compound (PMS)), K_2_SO_4_, ferrous sulfate (FeSO_4_ · 7H_2_O), Na_2_B_4_O_7_ · 10H_2_O, H_3_BO_3,_ sodium borohydride (NaBH_4_), and naproxen (C_14_H_14_O_3_, M_w_: 230.26 g/mol) were all obtained from Sigma-Aldrich (Saint Louis, MI, USA). Sodium chloride was obtained from AkzoNobel (Amsterdam, the Netherlands). The hollow fibers were provided by NX Filtration (Amsterdam, the Netherlands).

### 2.2. Fabrication and Surface Characterizations of Catalytic Membranes with Fe^0^ on or in the Selective Layer

Layer-by-layer assembly was used to build polyelectrolyte multilayers on top of the hollow fiber support membranes. In a typical coating process, the hollow fiber support membranes were first immersed in a polycation solution for 10 min and then rinsed in NaCl solution (500 mM) for 5 min three times to wash away any loosely attached polyelectrolytes. Subsequently, the membranes were immersed in a polyanion solution for another 10 min, and then the same washing process was conducted. After that, one bilayer of polyelectrolytes was coated on the support membranes, and the membranes with the desired bilayer number could be obtained by repeating the procedures above [28]. In this work, PDADMAC was used as the polycation, and PAA and PSS were used as the polyanions. To in situ synthesize Fe^0^ on or in the membrane selective layer, the membranes with an outmost layer of PAA were rinsed in a solution of FeSO_4_ with a concentration of 0.18 mM [25]. After the Fe^2+^ ions complex with the carboxylic acids in PAA, the membranes were rinsed three times in deionized water for 5 min to wash away the unbound Fe^2+^ ions. Subsequently, the membranes were put into NaBH_4_ solution (0.36 mM) for 30 min to reduce Fe^2+^ ions on or in the membrane surface into Fe^0^ particles. By repeating the procedures of putting fibers into the Fe^2+^ solution and then reducing them with NaBH_4_, membranes with a varied amount of binding/reducing cycles were obtained. To observe the surface morphologies and check the elemental composition of the membranes fabricated, scanning electronic microscopy (SEM, JEOL JSM-7610LA, Tokyo, Japan) with an energy-dispersive X-ray spectroscopy device (EDS) was employed.

### 2.3. Measurements of Water Permeability, Naproxen Rejection, and Degradation Efficiency

The water permeability of the fabricated hollow fiber membranes was measured in a crossflow setup. Under a target pressure, the permeate within a certain time interval was collected and weighed, and the water permeability (*P*, L/m^2^/h/bar) can be calculated using Equation (1):

(1) $$P=\frac{V}{A\;\Delta t\;p}$$

where *V* (*L*) is the permeate volume, *A* (m^2^) is the membrane area, *p* (bar) is the pressure applied, and Δt (h) is the permeation time.

Due to the wide use of naproxen as an anti-inflammatory drug, it was chosen as the model micropollutant in this work. In the measurement of naproxen rejection, 1 L naproxen solution with a concentration of 2 mg/L (8.7 μmol/L) was used as the feed solution. To keep the pH of the naproxen solution the same after adding PMS, Na_2_B_4_O_7_ · 10H_2_O (2.5 mM) and H_3_BO_3_ (10 mM) were used as a buffer to control the pH of the naproxen solution at around 7.8 [29,30]. The naproxen rejection measurement started with 24 h of permeating naproxen solution through the membranes to make sure that the membranes reached an adsorption-desorption equilibrium of naproxen [31]. During this process, all the permeates were cycled back to the feed solution. After that, the initial naproxen rejection before adding PMS was measured by collecting solution samples from both the feed and permeate sides. The concentrations of naproxen were measured by HPLC (Dionex Ultimate 3000, Thermo Fisher Scientific, Massachusetts, US), and the conditions used for HPLC measurement can be found in our previous work [18]. The rejection of naproxen (*R*) can be calculated based on Equation (2):

(2) $$R=(1-\frac{c_{p}}{c_{f}})\times 100\%$$

where *c_p_* (mg/L) and *c_f_* (mg/L) are the concentrations in the feed and permeate solution, respectively. After measuring the naproxen rejection without PMS, 1 mM PMS was added to the feed solution to ignite the catalytic reactions. After one hour, 800 μL of the feed and permeate solutions were collected, and subsequently, 200 μL methanol was added immediately into the samples to quench the catalytic reactions. The naproxen rejection after adding PMS was also calculated based on Equation (2).

## 3. Results and Discussion

The Fe^2+^ binding/reducing processes were first conducted on top of the symmetric polyelectrolyte multilayers of PDADMAC/PAA, and the stability of the membranes obtained was tested. Furthermore, due to the limited thickness of the selective layer, the cycle number of the Fe^2+^ binding/reducing processes needed to perform AOPs was also investigated. To compare with the symmetric polyelectrolyte multilayers built in Section 3.1, the asymmetric polyelectrolyte multilayers were developed in Section 3.2, and the naproxen removals in both feed and permeate sides were explored.

### 3.1. The Effects of Fe^0^ Synthesized on Symmetric Multilayers

The in situ synthesis of Fe^0^ in and on symmetric polyelectrolyte multilayers has been widely explored [20,25,26]. However, little attention has been paid to the effects of the formed Fe^0^ particles on the resulting membrane permeability and selectivity. Here we study both the catalytic properties and the separation properties of PDADMAC/PAA-based multilayers, with one, two, and three Fe^2+^ binding/reducing cycles to embed Fe^0^ nanoparticles.

**Figure 1:**
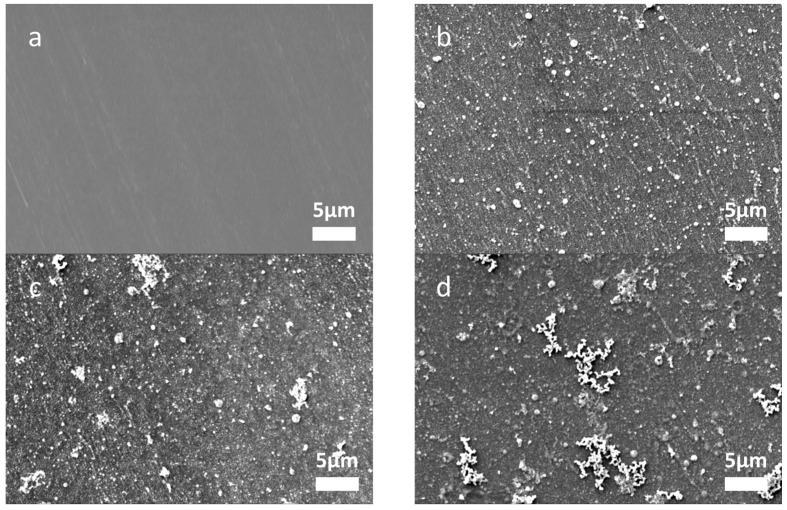
Surface morphologies of catalytic NF membranes with 0 (**a**), 1 (**b**), 2 (**c**), and 3 (**d**) Fe^2+^ binding/reducing cycles. The binding/reducing cycles were performed on top of hollow fibers with 4.0 bilayers of PDADMAC and PAA multilayers.

Figure 1 shows the surface morphologies of the membranes prepared with different Fe^2+^ binding/reducing cycles. The membrane without any binding/reducing cycles exhibits a neat surface, while due to the binding/reducing cycles, there are some small-sized particles on the other membrane surfaces. With the increase in binding/reducing cycles, more particles can be observed, and notably, aggregation of particles appears on the membrane surface when three binding/reducing cycles were conducted. To check if these particles on the membrane surface are Fe^0^ particles, the elemental compositions of the membranes with different binding/reducing cycles were confirmed by EDS. As shown in Table 1, when the binding/reducing cycles increased from one to three, the atom and mass percentages of iron elevated from 22.1% to 42.6% and 55.3% to 76.5%, respectively. These results correspond to the trends found in the literature [20], confirming that the amount of particles in and on the membrane surface can be effectively controlled by the amount of binding/reducing cycles.

**Figure 2:**
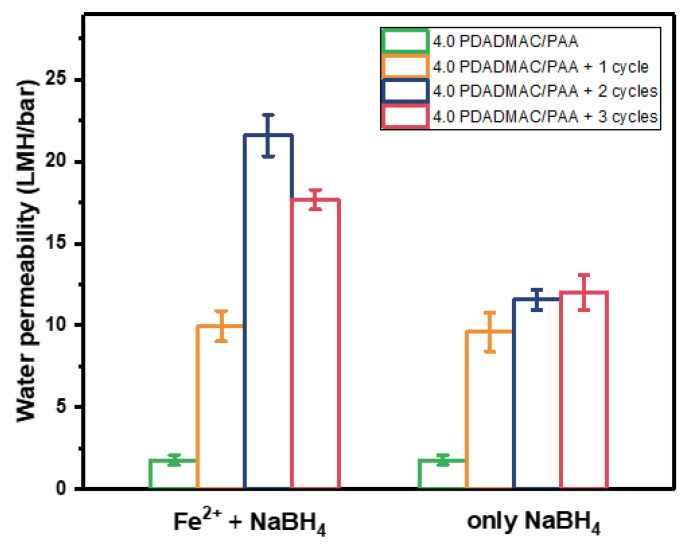
The comparison of the pure water permeability: the membranes with both Fe^2+^ binding and NaBH_4_ reducing process and the membranes with only NaBH_4_ rinsing procedure. The pressure was controlled at 2 bar. For every data point, three individual membrane samples were measured, and errors are given as the standard deviation.

To explore the effects of in situ synthesizing Fe^0^ particles on membrane performance, the permeabilities and the naproxen rejections of membranes with different binding/reducing cycles were measured. As shown in Figure 2, when the binding/reducing cycles increased from zero to three, the permeability measured with pure water elevated from 1.8 L/m^2^/h/bar to 17.7 L/m^2^/h/bar, indicating that the procedures of in situ synthesizing Fe^0^ particles make the polyelectrolytes multilayers more permeable, or even defective. To figure out if this increase in permeability is induced by the rinsing of NaBH_4_ or by the synthesized Fe^0^ within the multilayers, a series of membranes were fabricated by rinsing the hollow fibers with 4.0 bilayers of PDADMAC/PAA directly into NaBH_4_ without the binding procedure of Fe^2+^ ions. As shown in Figure 2, compared with the membranes of 4.0 bilayer PDADMAC/PAA without any binding/reducing cycles, the permeabilities of the membranes with only rinsing in NaBH_4_ all increased as well. Since the carboxyl group of PAA cannot be readily reduced by NaBH_4_ at room temperature [32], and PDADMAC is a strong polyelectrolyte, the likely explanations for this increased permeability are chain re-arrangements inside the PEM due to the strong alkalinity and the high ionic strength of the NaBH_4_ solution. At this high pH, PAA could become more completely charged, leading to additional charges in the layer, inducing additional swelling, while also the higher salinity could lead to more swelling [33,34,35]. As a consequence, higher permeabilities of the membranes are observed after rinsing in NaBH_4_. Besides the increase in permeability caused by NaBH_4_ rinsing, the permeabilities of the membranes with two and three Fe^2+^ binding/reducing cycles are still higher than the membranes with two and three rinsing cycles of NaBH_4_. The difference between the membranes with only NaBH_4_ rinsed, and the membranes with both Fe^2+^ binding and NaBH_4_ reducing is the Fe^0^ particles synthesized. This indicates that the synthesized Fe^0^ particles also contribute to the increase in water permeability, especially when more Fe^0^ particles are synthesized. Due to the penetration of Fe^2+^ ions into the polyelectrolyte multilayer when two and three binding/reducing cycles were performed, some Fe^0^ particles can be synthesized within the 4.0 bilayers of PDADMAC/PAA, resulting in more porous multilayers.

**Figure 3:**
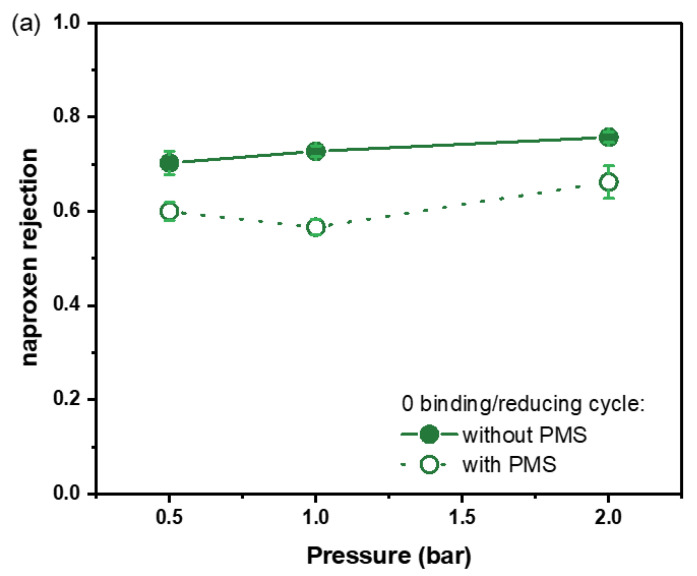

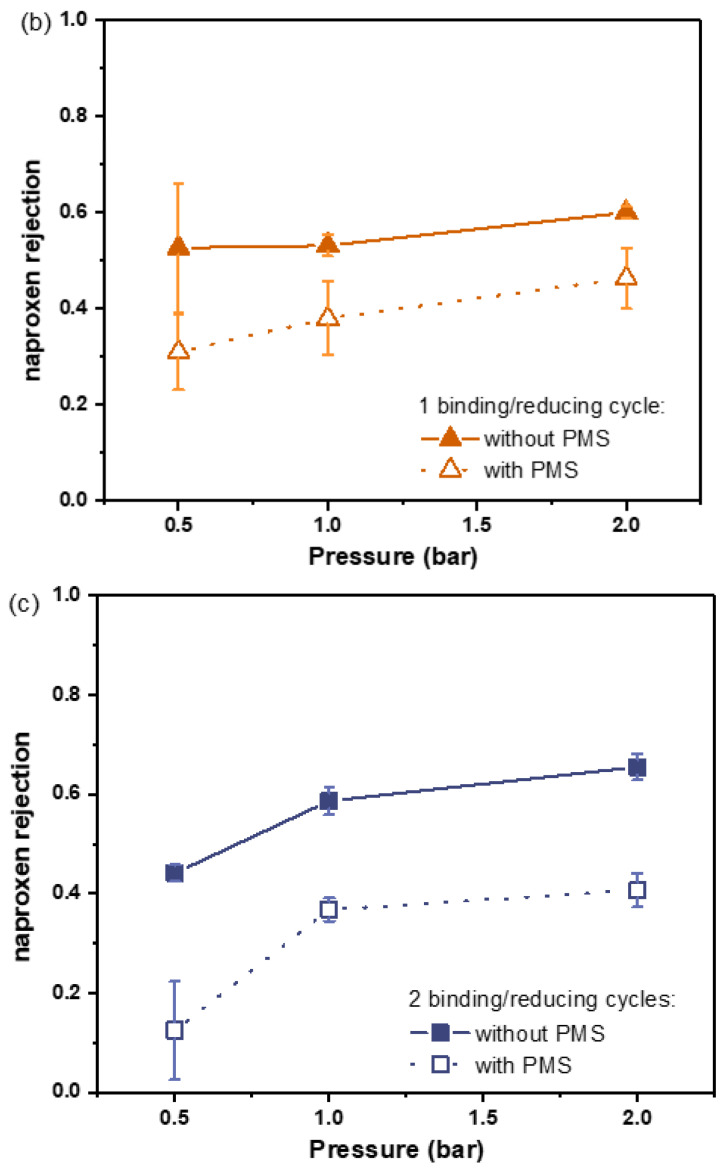

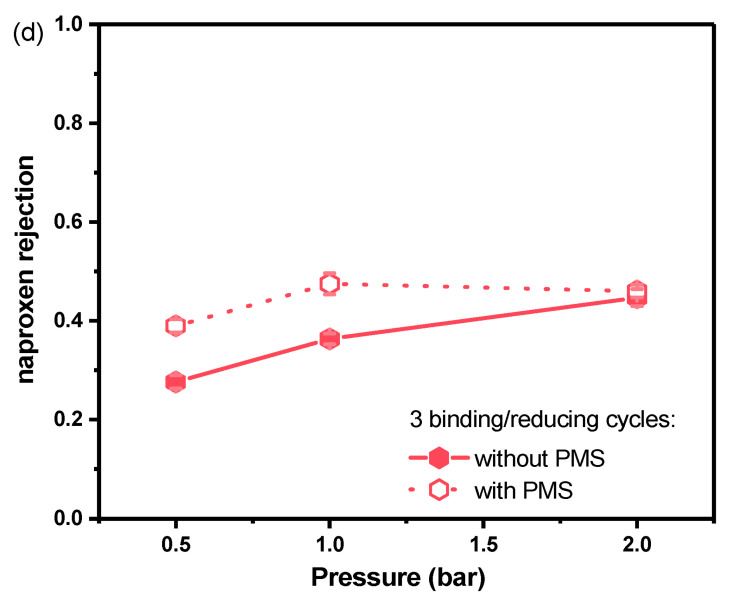
Naproxen rejection of catalytic NF membranes with 0 (**a**), 1 (**b**), 2 (**c**), and 3 (**d**) binding/reducing cycles. For every data point, three individual membrane samples were measured, and errors are given as the standard deviation.

The selectivity of the fabricated membranes was studied by measuring the naproxen rejections without adding PMS, which are shown in Figure 3. The membranes without any Fe^2+^ binding/reducing exhibited the highest naproxen rejections at different pressure, around 70%. With the introduction of Fe^0^ particles in polyelectrolyte multilayers, the rejection of naproxen decreased when more binding/reducing cycles were conducted, in other words, when more Fe^0^ particles were synthesized in polyelectrolyte multilayers. These naproxen rejections correspond to the higher water permeabilities of membranes with different Fe^2+^ binding/reducing cycles, showing that the polyelectrolyte multilayer of PDADMAC and PAA becomes less selective to naproxen after the in situ synthesis of Fe^0^ particles, likely due to increased swelling.

At the expense of the decreased selectivity of the polyelectrolyte multilayers, introducing Fe^0^ particles in polyelectrolyte multilayers is expected to improve naproxen removal after adding an oxidant. To investigate the degradation ability of the Fe^0^ catalysts embedded within the polyelectrolyte multilayers, PMS was chosen as the oxidant and added to the naproxen solution to start the AOP. As shown in Figure 3, surprisingly, the naproxen rejections of the membranes with zero, one, and two Fe^2+^ binding/reducing cycles all decreased after adding PMS. In this AOPs-coupled membrane separation process, the addition of PMS not only works as an oxidant for the reactions of AOPs but also leads to the increase in ionic strength, which can be the possible explanation for this drop in naproxen rejection [36]. To verify this hypothesis, a control experiment was performed, in which instead of 1 mM PMS, 1 mM K_2_SO_4_ was added to the naproxen solution to have the same ionic strength as 1 mM PMS. As shown in Figure S1, the naproxen rejection of the membrane with 1 mM K_2_SO_4_ decreased from 47% to 20%, indicating that the decline in naproxen rejection can be simply attributed to the change in ionic strength after adding PMS. However, there is still an improvement in naproxen rejection after adding PMS when the membranes with three Fe^2+^ binding/reducing cycles were measured, which is shown in Figure 3d. This implies that only with three Fe^2+^ binding/reducing cycles the amount of Fe^0^ catalysts is enough to overcome the decline of naproxen rejection caused by the increase in ionic strength, and an improvement in naproxen rejection can be observed. Moreover, it is also worth noting that the naproxen rejection after adding PMS slightly increased with the increase in pressure when one or two binding/reducing cycles were performed, which is opposite to what we found in our previous work [18]. This shows that with only one or two binding/reducing cycles, membrane separation is the dominant mechanism of naproxen rejection rather than the degradation via AOPs. However, when three binding/reducing cycles were conducted, as we can see in Figure 3d, the naproxen rejection with PMS slightly decreased when the pressure increased from 1 bar to 2 bar, indicating the influence of residence time. Furthermore, as shown in Figure S2, the permeabilities measured with naproxen solution and naproxen solution containing PMS are almost as same as the pure water permeability, indicating the chemical stability of this PDADMAC/PAA multilayer to withstand the harsh conditions during AOPs-based catalytic reactions.

In summary, Fe^0^ can be successfully synthesized in situ within the symmetric multilayers of PDADMAC/PAA, and the ability to degrade naproxen in an AOPs-coupled filtration process is also proved when three cycles of Fe^2+^ binding/reducing cycles are performed. However, the in situ synthesis method also induces a decrease in the selectivity of the polyelectrolyte multilayers, resulting from the rinse in an alkaline NaBH_4_ solution (0.36 M). Clearly, a PEM with higher chemical stability is required to withstand the harsh Fe^0^ synthesis conditions.

### 3.2. Asymmetric Selective Layer with Fe^0^ Catalysts

Since the in situ synthesis of Fe^0^ catalysts within the symmetric polyelectrolyte multilayers makes it less efficient in rejecting MPs, asymmetric polyelectrolyte multilayers were built by precoating 7.0 bilayers of PDADMAC/PSS below PDADMAC/PAA multilayers. As PDADMAC and PSS are both strong polyelectrolytes, the rinsing in alkaline NaBH_4_ would not be expected to affect the multilayers of PDADMAC and PSS [35]. Moreover, PDADMAC and PSS multilayers are expected to close the pores on the membrane surface, and thus, the in situ synthesis of Fe^0^ on PDADMAC/PAA multilayers can be conducted on a defect-free substrate [37]. As elaborated, the above 4.0 bilayers of PDADMAC/PAA offer Fe^2+^ ions the opportunity to penetrate the polyelectrolyte multilayers, resulting in the generation of Fe^0^ particles within the multilayers. Here, the effect of the bilayer number of PDADMAC/PAA deposited on the PDADMAC/PSS layer was also investigated by coating one, two, and four bilayers of PDADMAC/PAA, respectively. For all the membranes, three Fe^2+^ binding/reducing cycles were performed to ensure a sufficient amount of Fe^0^ catalysts.

**Figure 4:**
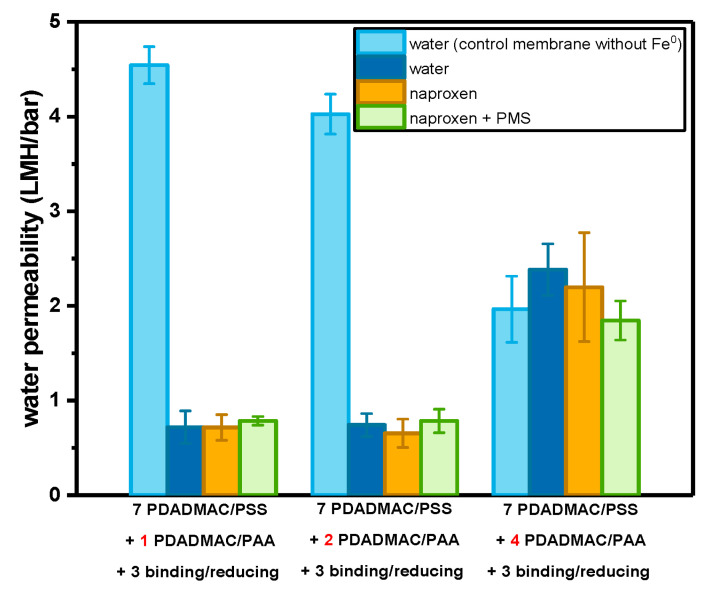
The comparison of the permeability of pure water, naproxen solution, and naproxen solution with PMS: 1.0, 2.0, and 4.0 bilayers of PDADMAC/PAA were coated on top of 7.0 bilayers of PDADMAC/PSS. A total of 3 binding/reducing cycles were conducted on top of the membranes. For every data point, three individual membrane samples were measured, and errors are given as the standard deviation.

**Figure 5:**
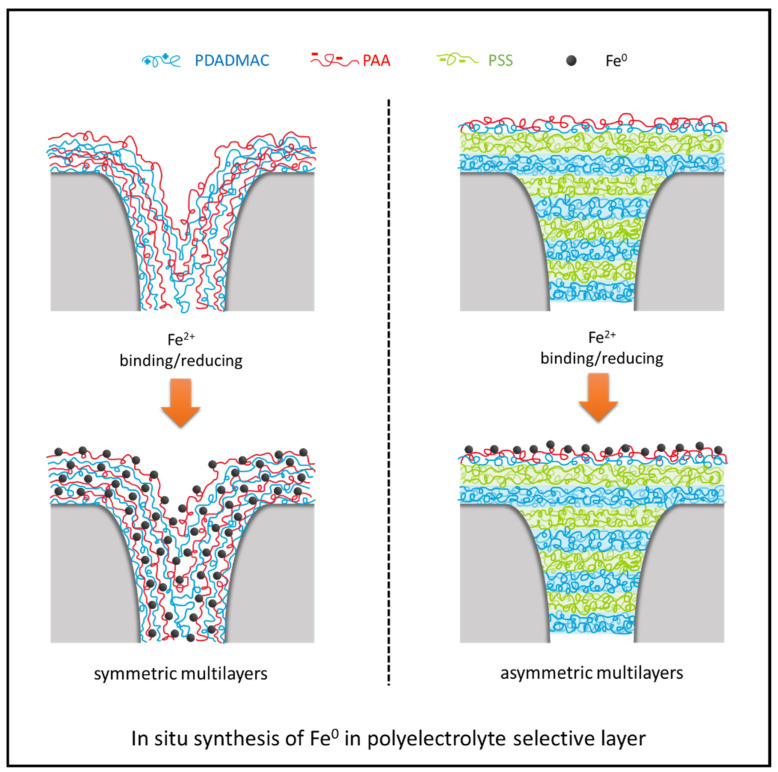
Schemes of in situ synthesizing Fe^0^ particles on symmetric polyelectrolyte multilayers (left) and asymmetric polyelectrolyte multilayers (right).

The surface morphologies of the membranes with different asymmetric selective layers are shown in Figure S3. Since all the membranes were fabricated with three cycles of Fe^2+^ binding/reducing, the surface morphologies of these membranes are quite similar to the membranes in Figure 1d, and no obvious difference can be observed. To further check the effect of the asymmetric structure and bilayer number of PDADMAC/PAA, the water permeability was measured, which is shown in Figure 4. The control membranes without any Fe^2+^ binding/reducing cycles exhibit a decrease in permeability with the increase in PDADMAC/PAA bilayers, indicating that the multilayers become denser with more coating of PDADMAC/PAA polyelectrolyte pairs. Once three Fe^2+^ binding/reducing cycles were conducted, the asymmetric membranes with four bilayers of PDADMAC/PAA exhibited an improvement of 20% in pure water permeability, which is much lower than the increase in water permeability (900%) when the in situ synthesis of Fe^0^ was performed on the symmetric multilayers of 4.0 PDADMAC/PAA bilayers (Figure 2). As shown in Figure 5, the negative effect of synthesizing Fe^0^ within the polyelectrolyte multilayers is mitigated by the 7.0 bilayers of PDADMAC/PSS, which added extra chemical resistance. Meanwhile, it can be observed that the permeability of membranes with one and two bilayers of PDADMAC/PAA shows an opposite trend, which decreased after the Fe^2+^ binding/reducing cycles. These different changes in water permeability after embedding Fe^0^ particles indicate that when one or two bilayers of PDADMAC/PAA were coated, the Fe^0^ particles were mainly synthesized on the surface of the PDADMAC/PAA bilayers, lowering the effective membrane area for filtration. While Fe^0^ particles are synthesized on 4.0 bilayers of PDADMAC/PAA, the penetration of Fe^2+^ ions into the multilayer leads to the generation of Fe^0^ particles within the multilayer, increasing the permeability of the multilayers.

**Figure 6:**
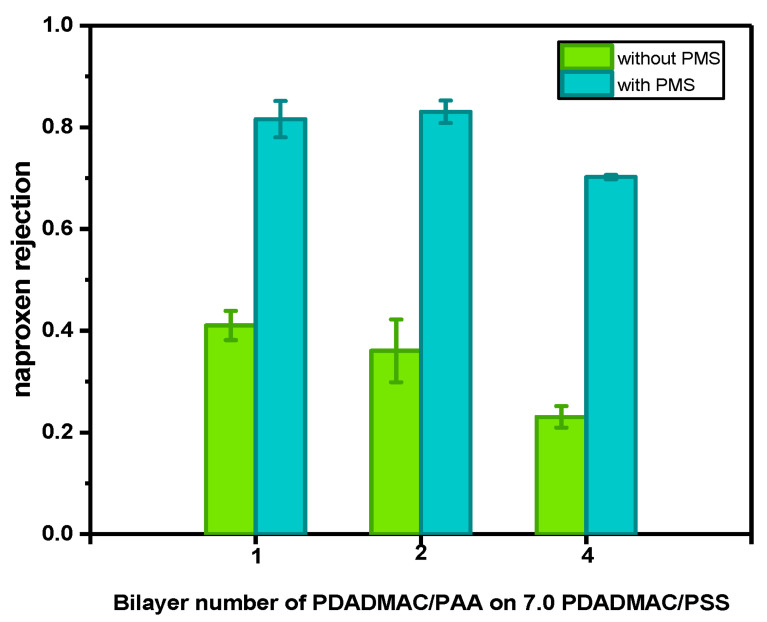
Naproxen rejection of catalytic NF membranes before and after the addition of PMS. The membranes were fabricated by coating 1.0, 2.0, and 4.0 bilayers of PDADMAC/PAA on top of 7.0 bilayers of PDADMAC/PSS. 3 Fe^2+^ binding/reducing cycles were then conducted on top of the membranes. All the membranes were measured under the flux of 1.4 ± 0.2 L/m^2^/h. For every data point, three individual membrane samples were measured, and errors are given as the standard deviation.

The naproxen rejections of the membranes with Fe^0^ particles were subsequently measured to check the selectivity of the membranes with an asymmetric selective layer. It needs to be mentioned here that all the membranes with an asymmetric selective layer were measured under the same flux to ensure the same residence time for the naproxen and PMS since the residence time has a significant influence on the degradation efficiency. However, as the permeabilities of the membranes with Fe^0^ particles in asymmetric multilayers are at least 10 times lower than the membranes with symmetric multilayers of 4.0 PDADMAC/PAA bilayers, the pressure needed is out of the limitation of our setup if we would have the same flux as in the measurement of the membranes with symmetric multilayers of 4.0 PDADMAC/PAA bilayers. So only the membranes with asymmetric selective layers were measured under the same flux, which is around 1.4 ± 0.2 L/m^2^/h, and a completely fair comparison of the naproxen rejection and degradation between asymmetric and symmetric membranes cannot be made here. As shown in Figure 6, the rejections of naproxen slightly decreased from 41% to 23% with the increase in PDADMAC/PAA bilayers from one to four, exhibiting the less dense structure of PDADMAC/PAA multilayers caused by the introduction of Fe^0^ particles. After the addition of PMS, all the membranes exhibit a significant enhancement in naproxen rejection due to the catalytic reactions on the membrane surface, an enhancement of almost 40%. This improvement in naproxen rejection here is much greater than in Figure 2, likely because of the higher residence time in these experiments. It needs to be emphasized that since layer-by-layer assembly of polyelectrolytes is a versatile method, polyelectrolyte multilayers with more bilayers or different polyelectrolyte pairs could be coated as alternatives to the PDADMAC/PSS used here. This would allow a higher selectivity to MPs, but the denser selective layer would also mean a higher energy consumption in the practical application.

Also, the naproxen concentration change in the feed solution was monitored. Normally, with the rejection of MPs, the concentration of MPs in the feed solution would increase, and a concentrated retentate solution could be formed, which would still need further treatment. As shown in Figure S4, a clear decrease in naproxen concentrations in the feed solution can be observed for all the membranes, which can be attributed to the contact of the feed solution and the Fe^0^ catalysts on the membrane surface. Meanwhile, with the decrease in the bilayer number of PDADMAC/PAA, more naproxen in the feed solution can be removed. When only one bilayer of PDADMAC/PAA was coated on top of 7.0 bilayers of PDADMAC/PSS, nearly 25% of naproxen in the feed solution can be degraded. This shows the major benefits of the catalytic NF membranes, which can effectively reduce the concentrated retentate solution.

## 4. Conclusions

A feasible method of in situ synthesizing Fe^0^ catalysts within polyelectrolyte multilayers was applied in this work to build AOPs-based catalytic membranes. With measurements of permeability, selectivity, and degradation efficiency of the membranes with symmetric multilayers of PDADMAC/PAA, it is revealed that the membranes become more open due to the harsh conditions during synthesis. For that reason, we propose the use of asymmetric polyelectrolyte multilayers, where the catalytic particles can be on top of a stable selective later. Indeed, through in situ synthesizing Fe^0^ particles on top of asymmetric polyelectrolyte multilayers consisting of 1.0 bilayer of PDADMAC/PAA on top of 7.0 bilayers of PDADMAC/PSS, the negative effect caused by the Fe^0^ catalysts within the selective layer can be mitigated and the obtained membranes exhibit over 80% naproxen rejection on the permeate side and 25% naproxen removal on the feed solution side. The in situ synthesis of Fe^0^ catalysts on the asymmetric polyelectrolyte multilayers provides a versatile method for building AOP-coupled membranes relevant to the treatment of MPs. With different polyelectrolyte pairs and varied bilayer numbers, it is possible to further improve the naproxen removal in both feed and permeate solution in future work. The high versatility of polyelectrolyte multilayer chemistry combined with process optimization has the potential to push this field forward.

## Figures and Tables

**Table 1:** Surface elemental composition of catalytic NF membranes with 0, 1, 2, and 3 Fe^2+^ binding/reducing cycles.

	Atom %			Mass %		
Element	C	O	Fe	C	O	Fe
0 cycle	72.0	27.7	0.3	65.2	33.3	1.5
1 cycle	62.9	15.0	22.1	33.9	10.8	55.3
2 cycles	54.4	12.4	33.2	24.1	7.3	68.6
3 cycles	47.3	10.1	42.6	18.3	5.2	76.5

## Data Availability

The data presented in this study are available in this article or Supplementary Materials here.

## Supplementary Materials

The following supporting information can be downloaded at: https://www.mdpi.com/article/10.3390/membranes13040388/s1 and there is no citation in the supplementary materials. Figure S1. The change in naproxen rejection of catalytic NF membranes with the addition of PMS or K_2_SO_4_; Figure S2. Permeability of catalytic NF membranes with different binding/reducing cycles; Figure S3. Surface morphologies of catalytic NF membranes:1.0 (a), 2.0 (b), and 4.0 (c) bilayers of PDADMAC/PAA were deposited on top of 7.0 bilayers of PDADMAC/PSS. 3 Fe^2+^ binding/reducing cycles were conducted; Figure S4. The naproxen removal in the feed solution. With the addition of PMS, the samples were taken from the feed solution after 1 h.
