# Supplementary material for: Advanced Oxidation Processes Coupled to Nanofiltration Membranes with Catalytic Fe0 Nanoparticles in Symmetric and Asymmetric Polyelectrolyte Multilayers
Source: Membranes (Basel). 2023 Mar 28;13(4):388. doi: 10.3390/membranes13040388 (PMC10141899; doi:10.3390/membranes13040388)
Supplement: Supplementary file 1 [file membranes-13-00388-s001.zip › membranes-2282682-supplementary.pdf]

Supplementary information

Advanced oxidation processes coupled to nanofiltration membranes with catalytic Fe^0 nanoparticles in symmetric and asymmetric polyelectrolyte multilayers

*Tao Wang[1], Enrique Serra Bachs,[1] Joris de Grooth[1], Wiebe M. de Vos[1]**

[1] University of Twente Institute for Nanotechnology: Universiteit Twente MESA+, The Netherlands

** Corresponding author: Wiebe M. de Vos*

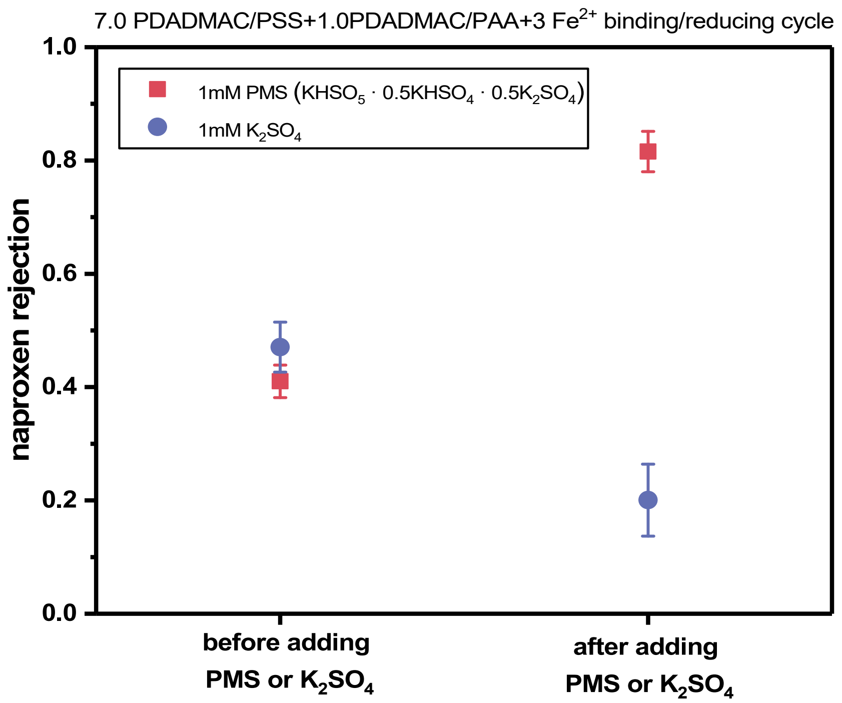

Figure S1. The change in naproxen rejection of catalytic NF membranes with the addition of PMS or K_2SO_4. The membrane was first coated with 7.0 bilayers of PDADMAC/PSS and then 1.0 bilayer of PDADMAC/PAA. 3 Fe^{2+} binding/reducing cycles were performed. The membranes were measured at 2.5 bar. For every data point, three individual membrane samples were measured and errors are given as the standard deviation.

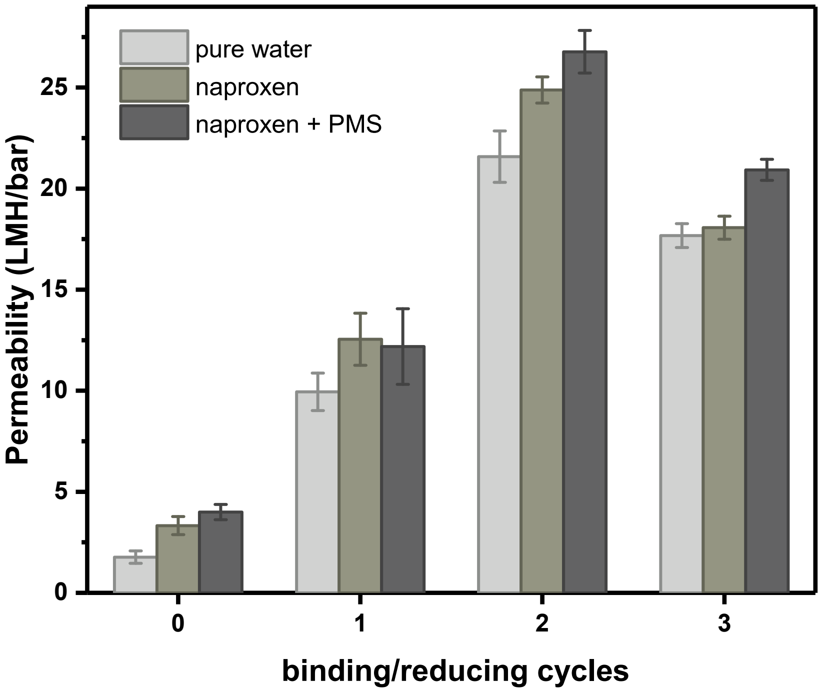

Figure S2. Permeability of catalytic NF membranes with different binding/reducing cycles. For every data point, three individual membrane samples were measured and errors are given as the standard deviation.

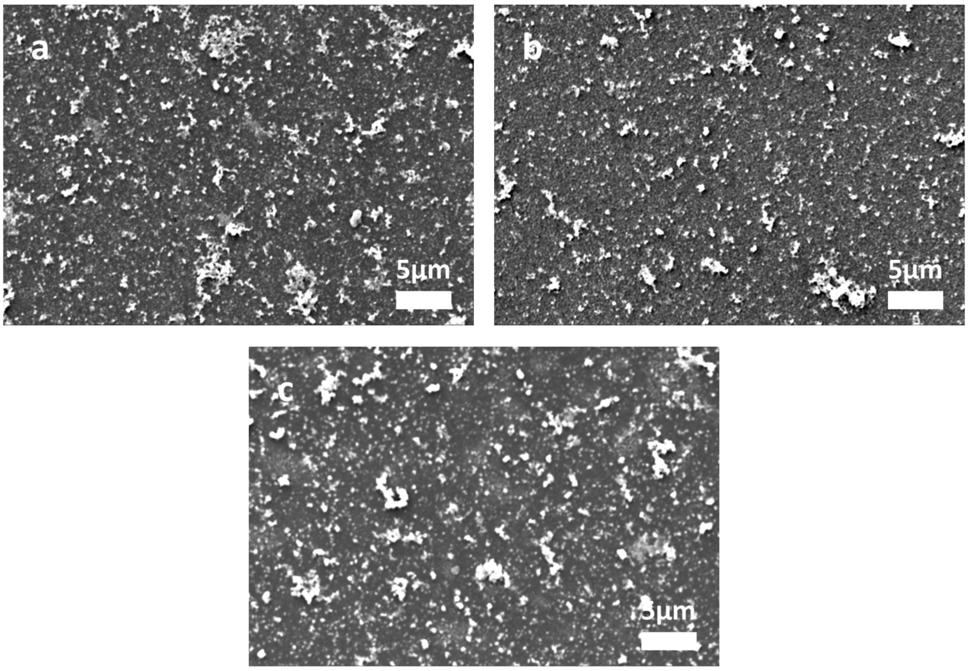

Figure S3. Surface morphologies of catalytic NF membranes:1.0 (a), 2.0 (b), and 4.0 (c) bilayers of PDADMAC/PAA were deposited on top of 7.0 bilayers of PDADMAC/PSS. 3 Fe^{2+} binding/reducing cycles were conducted.

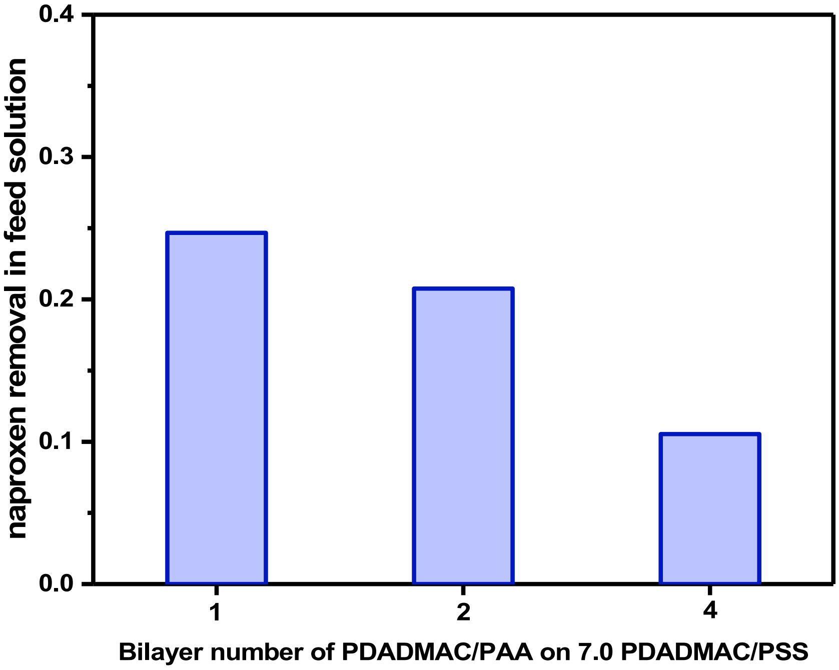

Figure S4. The naproxen removal in the feed solution. With the addition of PMS, the samples were taken from the feed solution after 1 hour. The membranes were fabricated by coating 1.0, 2.0, and 4.0 bilayers of PDADMAC/PAA on top of 7.0 bilayers of PDADMAC/PSS. 3 binding/reducing cycles were then conducted on top of the membranes.
